# Residential mobility and receipt of measles, mumps and rubella vaccination: analysis of linked primary care electronic health records in a disadvantaged London region

**DOI:** 10.23889/ijpds.v9i5.2640

**Published:** 2024-09-18

**Authors:** Nicola Firman, Laura North, Milena Marszalek, Marta Wilk, Ana Gutierrez, Rhodri Johnson, Lucy Griffiths, Rich Fry, Carol Dezateux

**Affiliations:** 1 Queen Mary University of London; 2 Swansea University

## Objective

Coverage for the measles, mumps and rubella (MMR) vaccination in north-east London (NEL) is the lowest in the United Kingdom. It has been hypothesised that children who move home frequently are less likely to receive vaccinations. We examined the association between number of addresses in the first two years of life, and receipt of MMR vaccination by age two.

## Approach

We derived households by pseudonymously linking the addresses of 178,028 children registered with a NEL general practitioner (GP) on their second birthday (2015-2021). We included 135,221 (76.0%) children (51.0% male) after excluding 42,807 with invalid household characteristics at age 24 months. The outcome was MMR vaccination by 24 months. Residential mobility was categorised by number of addresses (1, 2, ≥3) between earliest GP-registration and vaccination date/second birthday. We calculated the percentage receiving MMR by residential mobility and estimated adjusted odds (aOR; 95% confidence intervals [CI]) for MMR receipt.

## Results

105,392 (77.9%) children received MMR by age two, and 26,652 (19.7%) had more than one address. Receipt of MMR decreased with increasing residential mobility: 80.9% (95% CI: 80.6,81.1), 66.5% (65.9,67.1), and 58.6% (56.2,60.9) for children with one, two, or three or more addresses respectively. Children with multiple addresses were less likely to receive MMR by age two: aOR: 0.53 (0.51,0.55); 0.36 (0.32,0.40) for 2 and ≥3 addresses respectively.

## Conclusions

Uptake of MMR vaccine by two years of age is lower in children with multiple addresses. Further research is needed to identify actionable opportunities to improve MMR uptake in mobile populations.

